# A double knockout for *zinc transporter 8* and *somatostatin* in mice reveals their distinct roles in regulation of insulin secretion and obesity

**DOI:** 10.1186/s12263-024-00759-0

**Published:** 2024-11-20

**Authors:** Zhongyue Yang, Catherine P. Kirschke, Yimeng Cai, Liping Huang

**Affiliations:** 1 Graduate Group of Nutritional Biology, Department of Nutrition, University of California at Davis, One Shields Ave, Davis, CA 95616 USA; 2 USDA/ARS/Western Human Nutrition Research Center, 430 West Health Sciences Drive, Davis, CA 95616 USA; 3 Integrative Genetics and Genomics, University of California at Davis, One Shields Ave, Davis, CA 95616 USA

**Keywords:** Zinc transporter 8, ZnT8, Somatostatin, Glucose metabolism, Insulin and glucagon, Obesity and leptin

## Abstract

**Background:**

Both zinc transporter 8 (ZnT8) and somatostatin (Sst) play crucial roles in the regulation of insulin and glucagon secretion. However, the interaction between them in controlling glucose metabolism was not well understood. The aim of this study was to explore the interactive effects of a double knockout of *Znt8* and *Sst* on insulin and glucose metabolism in mice.

**Methods:**

Co-expression of ZnT8 with hormones secreted from gastrointestinal endocrine cells of mice was determined using immunofluorescence. Male *Znt8* knockout (*Znt8*KO), *Sst* knockout (*Sst*KO), double knockout for *Sst* and *Znt8* (DKO), and the wild-type (WT) mice were fed a regular chow diet (CD) or a high-fat diet (HFD) at 3 weeks old for 15 weeks. Weights and fasting or fed glucose levels were determined. Glucose and insulin tolerance tests were performed; metabolic-relevant hormone levels including insulin, glucagon, glucagon-like peptide 1, Pyy, and leptin were determined.

**Results:**

ZnT8 is co-expressed with Sst in a subpopulation of endocrine D cells in the gastrointestinal tract. The absence of ZnT8 expression resulted in an increased density of the dense cores in the secretory granules of the D cell. DKO mice had reduced weight compared to WT when maintained on the CD. Compared to *Znt*8KO and *Sst*KO, DKO mice did not show significant differences in fed or fasting blood glucose level regardless of dietary conditions. However, the CD-fed DKO mice had impaired insulin secretion without alterations in islet morphology or numbers. Moreover, DKO mice displayed diet-induced insulin resistance and disrupted secretion of metabolic-related hormones.

**Conclusions:**

Somatostatin as well as a normal insulin sensitivity are required for normalizing glucose metabolism in *Znt8*KO mice. ZnT8 may play a role in regulating fat mass and leptin secretion. These findings shed light on the multifaceted nature of *Znt8* and *Sst* interactions, opening new avenues to understand their roles in controlling glucose metabolism and fat mass.

**Supplementary Information:**

The online version contains supplementary material available at 10.1186/s12263-024-00759-0.

## Introduction

Zinc serves a pivotal role in insulin crystallization and glucose-induced insulin secretion in pancreatic β-cells. Approximately 70% of zinc ions in β-cells are sequestered within insulin-containing secretory vesicles [1, 2]. Two zinc ions bind to six insulin monomers forming a crystallized structure that enhances insulin storage capacity in β-cells [3]. The *SLC30A8* (*ZNT8*) gene in humans and rodents encodes a zinc transporter that is predominantly expressed in the β-cell of the pancreas [4]. It is a major zinc transporter delivering zinc ions into secretory granules for insulin crystallization in β-cells [4]. ZNT8 features six transmembrane domains with its N- and C-terminus both located on the cytosolic side of the secretory vesicle of the β-cell [5]. ZNT8 is also expressed in α-cells of the islets. Its expression level is correlating with glucagon concentrations in the human islets, hinting at its role in regulating glucagon secretion [6–8].

Genome-wide association studies (GWAS) in humans have identified a single nucleotide polymorphism (SNP, rs13266634) in *ZNT8* linking it to an increased risk of type 2 diabetes (T2D) [9]. Specifically, the risk C-allele (R325) is associated with an increased T2D risk in both European [10] and Asian populations [11, 12]. Subsequent GWAS have identified 12 nonsense mutations in *ZNT8* that produce various truncated forms of the ZNT8 protein [13]. For example, individuals carrying a loss-of-function mutation (rs200185429; p.Arg138* allele; c.412C > T) in *ZNT8* have reduced ZNT8 expression in β-cells due to haploinsufficiency of *ZNT8* that confers a significant protection (53%) against T2D [13, 14]. Studies in the genome-edited human induced pluripotent stem cell (iPSC)-derived β-like cells revealed that this protective effect was associated with improved proinsulin processing and glucose-induced insulin secretion via decrease in KATP channel activities and apoptosis of β-cells [13, 15, 16]. The same phenomena was also observed in the isolated human islets as well as in the humanized male mice carrying the SLC30A8 p.Arg138* mutation [13, 17, 18].

In mice, a global *Znt8* knockout (*Znt8*KO) led to an enhanced susceptibility to diet-induced obesity and glucose intolerance compared to the wild-type control (WT) [19, 20]. Zinc contents in secretory granules of the *Znt8*KO β-cells were significantly decreased [19]. *Znt8*KO mice also had reduced numbers of insulin-containing granules in beta-cells [21, 22]. However, it is worth noting that, although Z*nt8*KO decreases glucose-stimulated insulin secretion in mice, the physiological impact of *Znt8*-null on the whole-body glucose utilization varies, which is primarily dependent on the genetic background of the KO mice [23, 24].

Somatostatin (SST) is a peptide hormone secreted by the D cell located in many tissues, including the pancreatic islet, brain, stomach, and intestine [25–27]. SST is a well-known neuropeptide that is expressed throughout the brain as an inhibitory hormone, specifically inhibiting the release of the pituitary growth hormone [28]. SST is also present in the D cell of the gut mucosa as well as the myenteric neural plexus acting as a paracrine that negatively regulates hormone secretion in neighboring endocrine cells [29]. Through its paracrine actions, SST suppresses the secretion of ghrelin, glucagon-like-peptide 1 (GLP1), cholecystokinin (CCK), glucose-dependent insulinotropic polypeptide (GIP), gastrin, secretin, and vasoactive intestinal polypeptide [29, 30]. It also negatively regulates gastric acid secretion, gastric emptying, gallbladder contraction, and splanchnic blood flow [29, 30]. Additionally, SST functions as a paracrine in the pancreatic islet regulating glucagon and insulin secretion from α- and β-cells respectively, thus coordinating carbohydrate metabolism [31, 32].

Even though both ZNT8 and SST play important physiological roles in the regulation of insulin and glucagon secretion in the islet, the interaction between ZNT8 and SST in the control of glucose metabolism is not known. In the current study, we generated a double knockout for *Znt8* and *Sst* (DKO) to understand the crosstalk between them in the control of blood glucose levels and in the regulation of metabolic relevant hormone secretion under a chow-fed (CD) or a high-fat-fed (HFD) condition. We first examined the co-expression of ZnT8 with hormones secreted from enteroendocrine cells, including Sst, ghrelin, Glp1, Gip, and Cck using immunofluorescence microscopy. We also investigated the ultra-structure of the D cell in the pylorus of the stomach from *Znt8*KO and the WT littermate using electron microscopy, studying the effect of *Znt8*KO on the morphology of the D cell. We observed that ZnT8 was co-expressed with Sst in a subpopulation of D cells within the gastrointestinal tract, and the dense cores in the secretory granules of the D cells from *Znt8*KO mice appeared denser than WT. We also studied the markers for insulin and glucose metabolism and diet-induced obesity. Together, the results suggest that somatostatin as well as a normal insulin sensitivity are required for normalizing glucose metabolism in *Znt8*KO mice. ZnT8 may play a role in regulating fat mass and leptin secretion.

## Methods

### Ethical approval

All animal experiments were conducted in accordance with the National Institutes of Health Guidelines for the Care and Use of Experimental Animals, and animal protocols were approved by the Institutional Animal Care and Use Committee of the University of California, Davis (protocol numbers: #16467, #21973, and #23013).

### Mice and diets

*Znt8* heterozygous (*Znt8*HET) mice (*Slc30a8*^tm1a(KOMP)Wtsi^) in the genetic background of C57BL/6N were cryo-recovered from the KOMP Repository at the University of California, Davis (Davis, CA, USA). *Znt8* knockout (*Znt8*KO) mice and the WT littermates were generated from heterozygous mating. *Sst* heterozygous (*Sst*HET) mice (B6.N.129S4(129S6)-*Sst*^tm1Ute^/J; Stock No. 008117) were cryo-recovered from the Jackson Laboratories (Bar Harbor, Maine, USA). *Sst* knockout (*Sst*KO) mice and the WT littermates were obtained from heterozygous mating. *Znt8*HET and *Sst*HET mice were crossed to generate *Znt8*HET/*Sst*HET double heterozygous mice. Mice with a double knockout for *Znt8* and *Sst* (DKO) were produced by intercrossing *Znt8*HET/*Sst*HET siblings. All mice were housed in a temperature-controlled room at 22–24 °C with a 12-h light:dark cycle. Breeding mice were fed a standard laboratory chow diet (CD, Laboratory Rodent Diet 5001, LabDiet, Brentwood, MO) and double-distilled water ad libitum. Mice were genotyped at 2 weeks old. The experimental mice were weaned at 3 weeks old and randomly assigned to the CD or the HFD group (45% kcal fat; D12451, Research Diets, New Brunswick, NJ) for 15 weeks ad libitum. Body weight was measured weekly. At 14 and 17 weeks of age, non-fasting blood glucose levels were recorded at 8:00–9:00 in the morning using a Easy Touch® blood glucose meter (MHC Medical Products, LLC, Fairfield, OH). At 15 weeks of age, mice were fasted from 8:00 to 14:00, and 6-h fasting glucose levels were determined. At 16 weeks of age, mice were fasted for 4 h from 8:00 to 12:00, and an insulin tolerance test (ITT) was performed (see below for the details). At the end of the study (18 weeks of age), mice were fasted overnight (16–18 h) and an oral glucose tolerance test (OGTT) was conducted (see below for the details). The mice were then humanely euthanized in compliance with all institutional and national guidelines for the care and use of laboratory animals, under our approved protocols. Mice were first anesthetized with 100 mg/kg ketamine/10 mg/kg xylazine, and deep anesthesia was confirmed by the absence of the pedal reflex. Blood was collected via the submandibular route, followed by cervical dislocation to ensure a swift and humane euthanization. At necropsy, epididymal and retroperitoneal fat pads were isolated and weighed.

### Genotyping

Genotyping was performed as described previously using tail clips [30]. Briefly, genomic DNA was isolated from the mouse tail using a DNeasy Tissue Kit (Qiagen, Valencia, CA). PCR was used for genotyping. Primer information was given in Table 1 [31]. PCR products from *Znt8*HET mice yielded two distinct bands: a 573-bp DNA fragment (the *Znt8* KO allele) and a 376-bp DNA fragment (the WT allele) according to the protocol provided by the KOMP repository at the University of California, Davis. PCR products from *Sst*HET mice yielded two distinct bands: a 1000-bp DNA fragment (the *Sst* KO allele) and a 469-bp DNA fragment (the WT allele) according to the protocol provided by the Jackson laboratories. PCR products were detected by agarose-gel electrophoresis.

**Table 1:** Primers for genotyping the B6N.129S4(129S6)-*Sst*^tm1Ute^/J mouse strain^a^ and the Slc30a8^tm1a(KOMP)Wtsi^ mouse strain

Primer name	Forward primer sequence (5’ → 3’)	Reverse primer sequence (5’ → 3’)	Genotype
*Sst*-WT	TCAGTTTCTGCAGAAGTCTCTGGC	GAATGCCAATAGTTTGCG CAGCTCC	*Sst*WT
*Sst*-Mu	ATCCAGGAAACCAGCAGCGGCTAT	GAATGCCAATAGTTTGCG CAGCTCC	*Sst*KO
*Znt8*-WT	TCAAGATTCAGAATCAGTGTCATCTGG	AGACACCTGATCATGCATTTGCACC	*Znt8*WT
*Znt8*-KO	GAGATGGCGCAACGCAATTAAT	TGAATGTATGTGTGTGCATGTGTGGG	*Znt8*KO

Primer information was provided by the Jackson laboratories

^a^JAX stock #008117

### Immunofluorescence staining

The Pylorus of the stomach and duodenum of the small intestine were dissected at necropsy and placed in 4% paraformaldehyde solution made in 1 × PBS, pH 7.4, overnight. Preparation of paraffin embedded tissue, deparaffinization, rehydration, and subsequent sectioning (5 µm), and mounting were described prior [33, 34]. A guinea pig anti-mouse ZnT8 polyclonal antibody was generated with the peptide sequence CASRDSQVVRREIAKALSKSFTM by Genemed Synthesis Inc. (San Antonio, Texas). Other primary antibodies, including a rabbit polyclonal anti-human GLP1 (ab133329) and a rabbit monoclonal anti-human GIP antibody (ab209792), were purchased from Abcam (Waltham, MA). A rat monoclonal anti-human SST antibody (clone #906552) was bought from R&D Systems (Minneapolis, MN). All primary antibodies were diluted in 1 × PBS containing 2% mouse and 2% goat sera at 1:1000 (ZnT8), 1:500 (GLP1), 1:100 (GIP), and 1:25 (SST) for immunofluorescence staining. Secondary biotinylated goat anti-guinea pig antibody, goat anti-rat, and goat anti-rabbit were purchased from Vector Laboratories (Burlingame, CA) and diluted at 1:200–250 according to the manufacturer’s instructions. Nuclei were stained with DAPI (Vector Laboratories). Photomicrographs were obtained using an EVOS imaging system (ThermoFisher Scientific, Carlsbad, CA).

### Immunohistochemical analysis

Pancreases were dissected from 8-week-old *Znt8*KO, *Znt8*^+/-^, and WT mice at necropsy and placed in 4% paraformaldehyde solution made in 1 × PBS, pH 7.4, overnight. Preparation of paraffin embedded tissue, deparaffinization, rehydration, and subsequent sectioning (5 µm), and mounting were described previously [33, 34]. The anti-ZnT8 antibody was diluted at 1:1000 in 1 × PBS containing 2% mouse and 2% goat sera. A secondary biotinylated goat anti-guinea pig antibody (Vector Laboratories) was diluted at 1:200–250 in 1 × PBS containing 2% goat serum. Immunoperoxidase staining was accomplished using a Vectastain ABC kit and DAB substrate (Vector Laboratories). The immunoreactivity was developed in the tissue sections using a VECTASTAIN elite ABC kit and a HRP substrate ImmPACT DAB kit (Vector Laboratories) which produced brown deposits in the cells. Sections were counterstained with hematoxylin. Photomicrographs were acquired using an EVOS imaging system.

### Histological analysis and quantification of islet mass

﻿Tissue preparation and sectioning of the pancreas were as described in the Immunohistochemical analysis section above. Hematoxylin & eosin (H&E) staining was performed according to a published protocol [35]. Photomicrographs of whole pancreatic sections were acquired using an EVOS imaging system. The area of each pancreas section and the size of each islet was measured using ImageJ software [36]. Islet numbers in each pancreas section were counted. In addition, the area of the pancreas and the area of each islet were determined (*n* = 3–11/group). Islet/pancreas ratio (%), area of the islets per pancreas, and the islet number for each pancreas were obtained.

### X-gal staining for β-galactosidase

The pancreas of *Znt8*^+/-^ or WT mice (8 weeks old) was dissected at necropsy, rinsed in ice-cold 1 × PBS (pH 7.4), and placed in ice-cold 10% formalin overnight at 4 °C. The pancreas was washed with 1 × PBS and placed in ice-cold 30% sucrose overnight at 4 °C. It was then embedded in an optimal cutting temperature compound (O.C.T., Sakura Finetek USA, Inc., Torrance, CA) in a liquid nitrogen–chilled isopentane bath, followed by sectioning in 10-μm thicknesses and allowed to dry overnight at room temperature (RT). The sections were subsequently stained for β-galactosidase activity as described prior [37]. The sites of β-galactosidase activities were stained blue, and the nuclei were stained pink.

### Electron microscopy

The Pyloric tissues from 8-week-old *Znt8*KO and WT mice were collected and fixed using Karnovsky's fixative in 0.1 M sodium phosphate buffer (Sorenson's). The section process was performed as described previously [38]. The sections were viewed on a Phillips CM120 Biotwin (Hillsboro, OR). Digital images were taken with a Gatan BioScan model digital camera (Pleasanton, CA).

### Oral Glucose Tolerance Test (OGTT)

Before the test, mice were fasted for 16–18 h. On the test date, mice were weighed, and fasting glucose levels were determined using an Easy Touch® blood glucose meter. Glucose (20% glucose solution (wt/wt) in 1 × saline sterilized by a 0.2 µm filter) was administered (1.5 g/kg) directly into the stomach of mice via oral gavage. During the test, blood was collected from the submandibular vein at 0, 15, 30, 60, and 120 min before and after the glucose administration. Blood glucose levels were determined using the glucose meter. Plasma was then purified by centrifugating the blood samples at 960 g at 4 °C for 10 min and stored at -80 °C until use.

### Intraperitoneal Insulin Tolerance Test (ITT)

Before the test, mice were fasted for 4 h (07:30–11:30) and weighed. After the baseline glucose levels were determined, mice were intraperitoneally injected with Humulin® R U-100 (5.5 U/kg; Eli Lilly, Indianapolis, IN). Glucose levels were determined from the blood samples collected from the mouse tail vein at 0, 30, 60, and 120 min after the insulin administration using an Easy Touch® blood glucose meter.

### Determination of plasma metabolic-related hormone levels

Plasma insulin, glucagon, Glp1 (total), leptin, and Pyy (total) levels were determined by a mouse U-PLEX Diabetes Combo 1 kit (MSD, Rockville, MD) using a Sector Imager 6000 instrument according to manufacturer’s instruction (MSD).

### Statistical analysis

Results are presented as mean ± SEM. A one-way or two-way ANOVA test was used to compare the means of multiple groups when appropriate followed by a post-hoc test (Tukey’s test) to identify specific and statistical differences between individual dietary or genotypic groups. Differences were significant at *p* < 0.05.

## Results

### Co-expression of ZnT8 with Sst in a subset of the D cells in the mouse pylorus and duodenum

Genome-wide association studies revealed that allelic *ZNT8* insufficiency in humans is associated with reduced risk of T2D by ~ 60% [13]. Thus, we hypothesized that reduced or lack of ZNT8 expression, which negatively affects glucose-stimulated insulin secretion but not blood glucose levels [39], might trigger a compensatory action of other metabolic relevant hormone productions or releases to normalize glucose metabolism. Such metabolic hormones include Gip and Glp1, the two gut hormones that are responsible for stimulation of about 50–60% of postprandially secreted insulin from the pancreas, leading to improved glucose utilization in the body [40, 41]. ZnT8 deficiency might influence Sst function, an inhibitory regulator of Glp1 and Gip secretion, thus coordinating glucose metabolism.

**Fig. 1:**
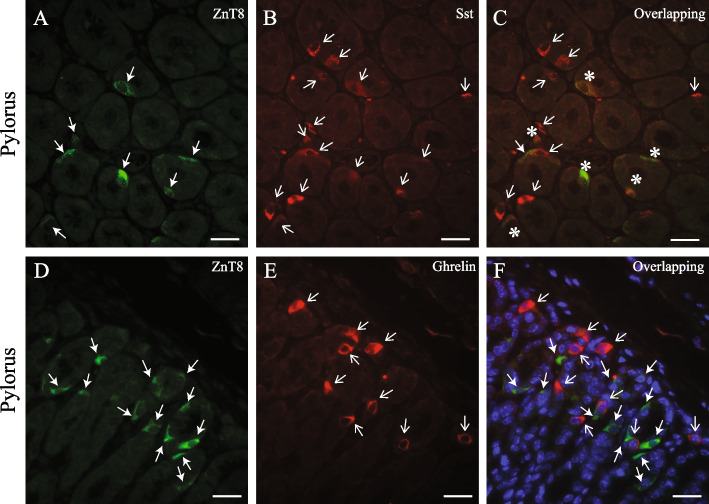
Immunofluorescence study of ZnT8, Sst, and ghrelin in the pylorus of the mouse stomach. ZnT8 (green, **A** & **D**) was co-stained with Sst (red, **B**) or ghrelin (red, **E**). ZnT8, Sst, or ghrelin was detected in the cytoplasm of endocrine cells. Solid arrows indicate the cells stained with ZnT8 (**A** & **D**). Open arrows indicate the cells stained either Sst (**B**) or ghrelin (**E**). Nuclei were stained with DAPI in blue (**F**). Scale bar = 25 µm. We detected that a fraction of Sst-expressing endocrine cells in the pylorus co-expressed with ZnT8 (asterisks) (**C**). However, we did not observe co-expression of ZnT8 with ghrelin in the ghrelin-expressing endocrine cells in the merged images (**F**). Staining was repeated 2–3 times in the pyloric tissues isolated from the wild type B6 mice. ZnT8, zinc transporter 8 or Sla30a8; Sst, somatostatin

**Fig. 2:**
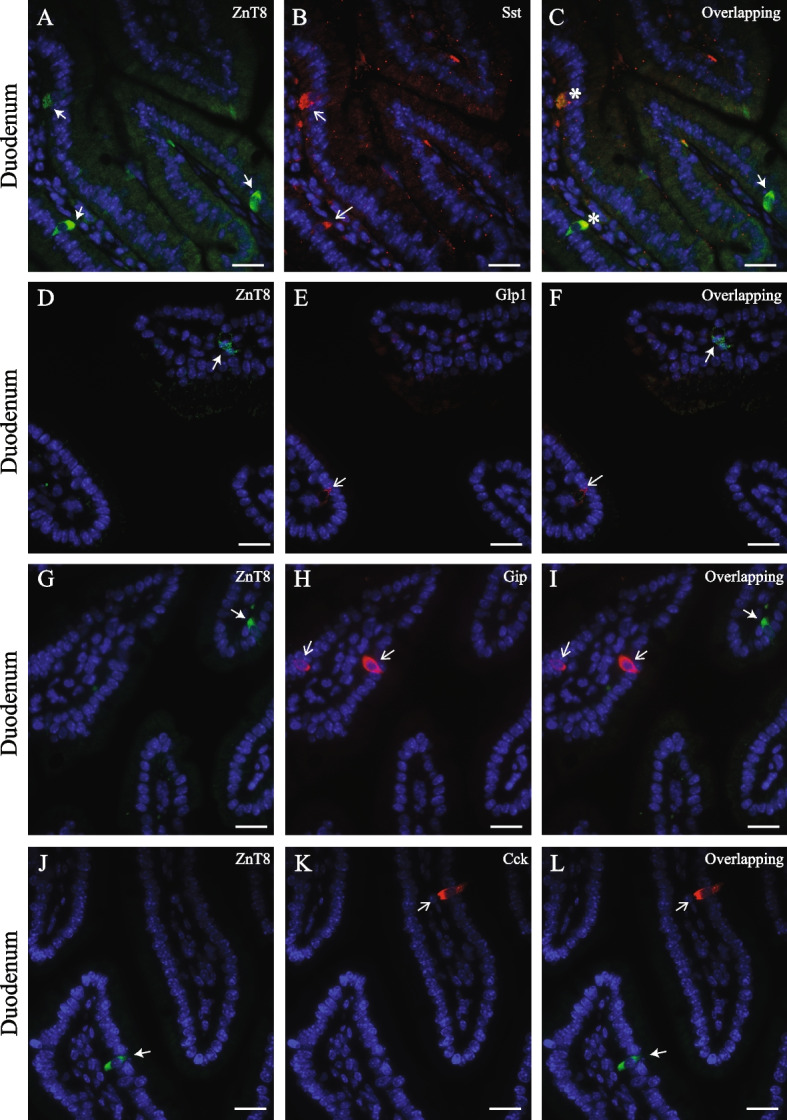
Immunofluorescence study of ZnT8, Sst, Glp1, Gip, and Cck expressed in the duodenum of the mouse small intestine. ZnT8 (green, **A**, **D**, **G**, & **J**) was co-stained with Sst (**B**), Glp1 (red, **E**), Gip (red, **H**), or Cck (red, **K**). ZnT8, Sst, Glp1, Gip, or Cck was detected in the cytoplasm of endocrine cells. Solid arrows indicate the cells stained with ZnT8 (**A**, **D**, **G**, & **J**). Open arrows indicate the cells stained Sst (**B**), Glp1 (**E**), Gip (**H**) or Cck (**K**). Asterisks indicate the endocrine cells co-stained ZnT8 with Sst (**C**). Nuclei were stained with DAPI (blue). Scale bar = 25 µm. We did not observe a co-expressing pattern of ZnT8 with Glp1 (**F**), Gip (**I**), or Cck (**L**) in the endocrine cells of the duodenum in the merged images. Staining was repeated 2–3 times in the intestinal tissues isolated from the wild type B6 mice. Sst, somatostatin, Glp1, glucagon-like peptide 1; Gip, glucose-dependent insulinotropic polypeptide; Cck, cholecystokinin

To investigate our hypothesis, we first determine whether ZnT8 was co-expressed with Sst, ghrelin, Glp1, Gip, or Cck in the enteroendocrine cells of the mouse gut mucosa using immunofluorescence microscopy. As shown in Fig. 1, in the pylorus of the stomach, we found that ZnT8 was co-expressed with Sst in a subpopulation (40–50% of the Sst-expressing cells) (A-C). A similar co-expression pattern of ZnT8 and Sst was observed in the duodenum of the small intestine (Fig. 2, A-C). This co-expression was detected using a monoclonal antibody generated from a full-length of human SST (Figs. 1 & 2) or a polyclonal antibody generated from a human SST peptide spanning the amino acids (aa) 50–116 (data not shown; ABIN6148498, Antibodies-online.com, Limerick, PA). On the other hand, the co-expression of ZnT8 and Sst was not evident in the mouse pylorus when a polyclonal antibody against the human SST generated from a SST peptide containing 25–107 aa (data not shown; PA5-82,678, RRID#AB_2789834, ThermoFisher Scientific), suggesting that ZnT8 is co-expressed in the D cells expressing the C-terminal end of the SST peptide where both SST 14 (SS-14) and 28 (SS-28) amino acid isoforms are located. Remarkably, we did not detect any overlapping expression of ZnT8 with ghrelin in the pylorus (Fig. 1, D-F) and Glp1 (Fig. 2, D-F), Gip (Fig. 2, G-I), or Cck (Fig. 2, J-L) in the duodenum. Together, these results suggested that ZnT8 may interplay with Sst and indirectly regulate Glp1, Gip or other gut hormone secretion.

### Generation and characterization of *Znt8*KO mice

**Fig. 3:**
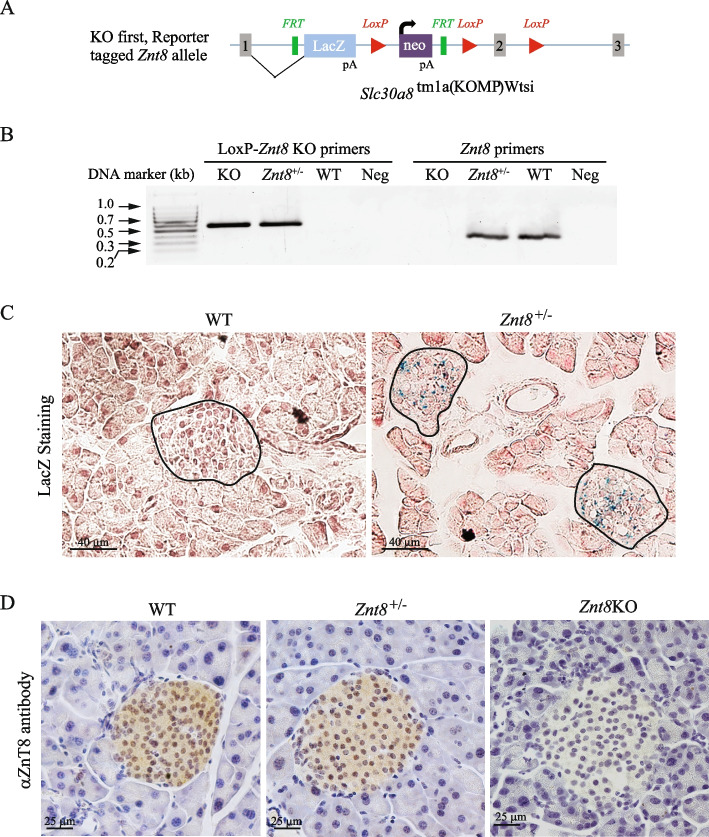
Generation of *Znt8*^+/-^ (*Slc30a8*^tm1a(komp)wtsi^) mice. **A **The construct. A promoter-driven targeting cassette was inserted into the mouse *Znt8* gene between exons 1 and 2. The amino acids encoded from exon 1 (MEFLERTYLVNDQATKMYAFPLDRWTRCWESGYPH) were fused in frame with the reporter LacZ sequence followed by a poly A signal in the *Znt8*KO mice. Gray boxes, *Znt8* exons; FRT, flippase recognition target; LoxP, Lox sequences (short target sequences recognized by the Cre recombinase) derived from bacteriophage P1; LacZ, the bacterial β-galactosidase gene; Neo, the neomycin resistant gene. Arrow indicates the transcription start site for the neomycin resistant gene. **B **The image of genotyping results for *Znt8*^+/+^, *Znt8*^+/-^, and *Znt8*^−/−^. Genomic DNA was genotyped by 2 sets of primer pairs, LoxP-*Znt8*KO and *Znt8* primer pairs that detected the mouse genomic DNA with or without the targeting cassette, respectively. If a DNA fragment (573-bp) was amplified by PCR using the LoxP-*Znt8*KO primer pair, it indicated the *Znt8*^−/−^ genotype. If a 376-bp DNA fragment was detected using the *Znt8* primer pair, it indicated *Znt8*^+/+^ genotype. Lastly, if two DNA bands were detected (573- and 376-bp), it indicated *Znt8*^+/-^ genotype. The negative control (Neg) is shown (PCR reactions without the genomic DNA templates). DNA marker is shown on the left. **C **LacZ staining in the mouse pancreas. Cryo-sectioned pancreas (*Znt8*^+/-^) was stained with X-gal. The LacZ activity (blue) was only detected in the islets of *Znt8*^+/-^ but not wild type (WT) mice. **D **Immunohistochemical staining of ZnT8 in the mouse pancreases. Representative images of ZnT8 staining are shown. ZnT8 protein (brown color) was detected in the islets of WT and *Znt8*^+/-^ mice. Staining was absent in the islets of *Znt8*^−/−^ mice. The scale bar = 25 µm. Staining was repeated 2–3 times per genotype

To further investigate whether Sst is involved in the ZnT8 deficiency-induced complementary action of gut hormones in normalizing glucose metabolism, we crossed a *Znt8* knockout (*Znt8*KO) mouse strain (*Slc30a8*^tm1a(KOMP)Wtsi^) to a *Sst*KO strain (the Jackson Laboratories, stock No. 008117) and characterized the resulting male mice with indices of insulin and glucose metabolism under either a normal chow dietary condition or a high-fat dietary challenge. Both *Znt*8KO and *Sst*KO were maintained in the C57BL/6NJ genetic background. Since the *Slc30a8*^tm1a(KOMP)Wtsi^ mouse strain had not been characterized and reported previously, we first examined the strain for ZnT8 expression and the knock-in β-galactosidase activities. *Znt8*KO or *Znt8* heterozygous (*Znt8*^+/-^) mice were expected to have a β-galactosidase gene in the *Znt8* locus (Fig. 3A) and could be readily identified by PCR genotyping (Fig. 3B). As expected, the LacZ activity (blue) was only detected in the islets from *Znt8*^+/-^ mice but not from the WT mice (Fig. 3C). Likewise, we detected the expression of ZnT8 in the islets from the WT and *Znt8*^+/-^ mice, but not from the *Znt8*KO mice (Fig. 3C).

### Effect of *Znt8* deficiency on the granule morphology in the D cell

**Fig. 4:**
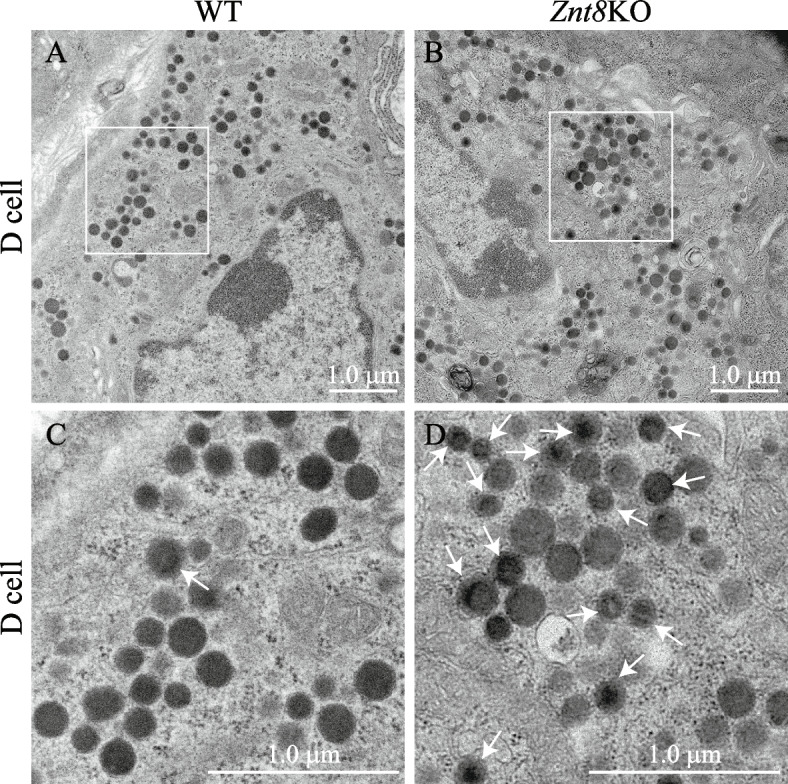
Representative electron micrographs of the D cells in the pylorus of the mouse stomach isolated from *Znt8*KO and WT mice. **A** and (**B**), low-magnification images of electron micrographs. **A **A WT D cell. **B **A *Znt8*KO D cell. The cytoplasm of both WT and *Znt8*KO D cells are well granulated with various sizes. **C** and **D**, high-magnification images of electron micrographs. **C **Secretory granules in the WT D cell. The secretory granules are in various sizes with a homogenous moderate density. **D **Secretory granules in the *Znt8*KO D cell. Compared to the WT granules (**C**), the secretory granules in the *Znt8*KO D cell exhibit more heterogenous electron densities with circular or oval profiles within the granules (solid arrows). Scale bars = 1.0 μm. The electron microscopy was done twice in the pyloric tissues isolated from the wild type B6 mice

ZnT8 is a major zinc transporter for carrying zinc ions into the insulin-secreting granules in β-cells [4]. Deletion of *Znt8* alleles alters the morphology of the insulin-containing granules in the mouse β-cells with increased amounts of granules having empty, light core, or rod-shaped core [7]. In the present study, we showed that ZnT8 was expressed in Sst-positive D cells in the pylorus and duodenum (Figs. 1 & 2). Thus, it is conceivable that *Znt8* KO might also alter the morphology of granules in D cells. Using electron microscope analysis, we observed increased densities of the dense cores in the secretory granules accompanied with an increased number of atypical granules with rod-shaped cores in the D cells from the *Znt8*KO pylorus. Whereas granules from the WT D cells in the pylorus were of moderate density with a somewhat homogenous distribution pattern (Fig. 4). However, the granule numbers and shape seemed unaffected by the *Znt8* knockout in these D cells.

### Effects of *Znt8* deficiency on body weight, insulin secretion, and glucose metabolism

**Fig. 5:**
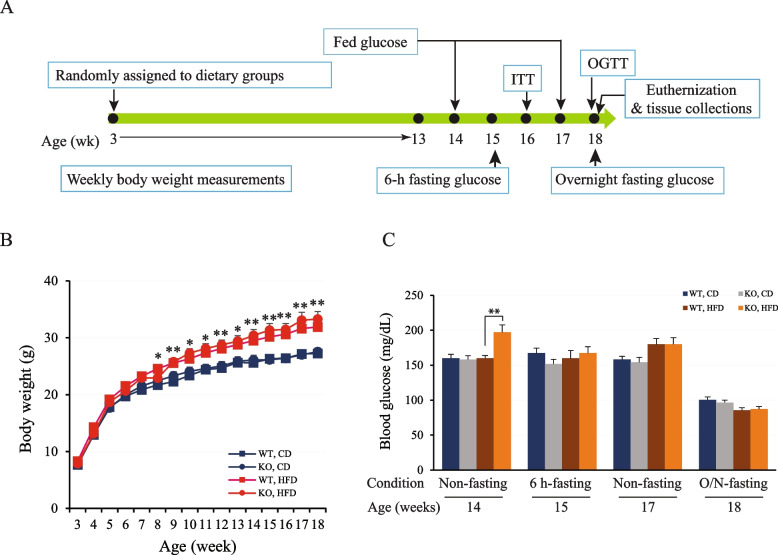
Growth and glucose levels in *Znt8*KO and WT male mice. **A **Scheme of dietary feeding and experiments during the study. **B **Body weight (g) of male *Znt8*KO and WT mice aged from 3 to 18 weeks fed either a regular chow diet (CD) or a high-fat diet (HFD). Both *Znt8*KO and WT mice fed the high-fat diet started gaining significant weight compared to the respective control diet group (*, *p* < 0.05); **, *p* < 0.01). However, there was no difference (*p* > 0.05) in weight gain between the two genotypic groups within the same dietary group. **C **Fasting and non-fasting blood glucose levels over the dietary challenge period. ** (*p* < 0.01) indicates the significant difference in non-fasting glucose levels (mg/dL) between 14-week-old male mice of *Znt8*KO and WT in the HFD group. All data are presented as mean ± S.E., *n* = 8–10/group. Body weight and blood glucose data were analyzed by a one-way or two-way ANOVA test to compare the means of multiple groups when appropriate followed by a post-hoc test (Tukey’s test). CD, chow diet; HFD, high-fat diet

To assess this new *Znt8*KO strain regarding insulin secretion and glucose metabolism, male *Znt8*KO and WT littermates were fed a CD or a HFD (45% kcal fat) for 15 weeks. The HFD was introduced when male mice were at weaning (3 weeks old). The progress of diet-induced obesity, insulin resistance, or glucose intolerance was monitored by body weight, fasting or non-fasting blood glucose levels, insulin tolerance test (ITT), and oral glucose tolerance test (OGTT) (Fig. 5A). At 3 weeks of age, no significant difference (*p* > 0.05) in body weight was observed between *Znt8*KO and WT mice (*Znt8*KO, 8.0 ± 0.2 g, *n* = 17, vs WT, 7.9 ± 0.3 g, *n* = 18). After 15 weeks on HFD, both *Znt8*KO and WT mice gained significant weight (*Znt8*KO, 19.7 ± 0.7 g vs WT, 19.6 ± 0.6 g) at a similar rate compared to the respective genotype in the CD group (*p* < 0.01) (Fig. 5B). Moreover, no significant changes in weight were observed between *Znt8*KO and WT mice in the same dietary group (*p* > 0.05) (Fig. 5B). After 11 weeks on HFD, male *Znt8*KO mice (14 weeks old) presented a slightly higher non-fasting blood glucose level than the WT control (*Znt8*KO, 197.3 ± 10.6 mg/dL, *n* = 8 vs WT, 159.9 ± 4.0 mg/dL, *n* = 8; *p* < 0.01). However, this difference was no longer detectible when the KO mice were at 17 weeks old (*Znt8*KO, 180.3 ± 9.2 mg/dL, *n* = 8 vs WT, 180.3 ± 7.9 mg/dL, *n* = 8; *p* > 0.05). We did not observe any difference in fasting glucose levels between *Znt8*KO and WT mice, including 6-h fasting levels (*Znt8*KO, 167.5 ± 10.6 mg/dL, *n* = 8 vs WT, 160.0 ± 11.1 mg/dL, *n* = 8) and overnight fasting levels (*Znt8*KO, 87.3 ± 3.6 mg/dL, *n* = 8 vs WT, 85.6 ± 3.6 mg/dL, *n* = 8) after 12 and 15 weeks on HFD, respectively. Neither did we detect difference in fasting or non-fasting blood glucose levels between *Znt8*KO and WT mice fed CD. Taken together, male *Znt*8KO mice was associated with an increased non-fasting blood glucose level at early age after exposure to the high-fat diet.

**Fig. 6:**
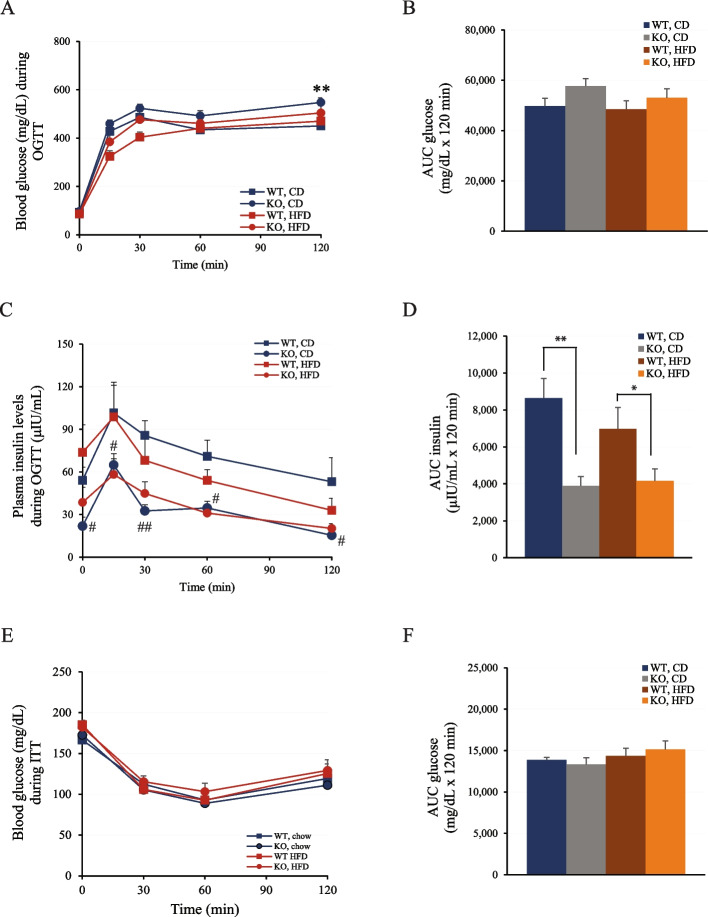
Oral glucose tolerance test (OGTT) and intraperitoneal insulin tolerance test (ITT) in male *Znt8*KO and WT mice. **A **Blood glucose levels during OGTT. *Znt8*KO mice fed CD had a significant increase in the blood glucose level at 120 min post the glucose load, compared to the WT control in the same dietary group (**, *p* < 0.01). **B **Area Under the Curve (AUC) of blood glucose levels during OGTT. No significance was found between the genotypic groups fed either diet. Fifteen-weeks HFD challenge had a limited effect on the AUC of blood glucose levels of either the *Znt8*KO or WT mice during OGTT. **C **Plasma insulin levels during OGTT. *Znt8*KO mice fed CD had significantly lower plasma insulin levels at 0 min before oral glucose challenge and 15, 30, 60, and 120 min after the challenge than the WT control in the same dietary group. #, *p* < 0.05; ##, *p* < 0.01. **D **AUC of plasma insulin levels during OGTT. *, (*p* < 0.05) or **, (*p* < 0.01) denotes significant difference between *Znt8*KO and WT mice within the same dietary group. **E **Blood glucose levels during ITT. **F **AUC of blood glucose levels during ITT. No significance was found between the genotypic groups fed either diet. All data are presented as mean ± S.E., *n* = 8–10/group. Blood glucose and plasma insulin data were analyzed by a one-way or two-way ANOVA test to compare the means of multiple groups when appropriate followed by a post-hoc test (Tukey’s test). CD, chow diet; HFD, high-fat diet

To investigate whether *Znt8* deficiency affected insulin secretion and/or glucose handling, an oral glucose tolerance test (OGTT) was performed and insulin concentrations during OGTT were determined. As shown in Fig. 6A, at 120 min after OGTT, the CD-fed *Znt8*KO mice had about 20% higher blood glucose level (548 ± 40 mg/dL, *n* = 10) than the CD-fed WT control (450 ± 19, *n* = 8). However, the area under the curves (AUC) for the glucose levels during OGTT were not significantly different between the CD-fed *Znt8*KO and WT mice (*p* > 0.05). Alternatively, we found that, compared to the WT control, plasma insulin levels (µIU/mL) during OGTT in the CD-fed *Znt8*KO mice was greatly reduced at all examined timepoints by 36–71% (Fig. 6C). The AUC for the insulin levels of the CD-fed *Znt8*KO mice during OGTT was reduced by 55% compared to the WT control (Fig. 6D, p < 0.01). A similar reduction trend was also observed in the HFD-fed *Znt8*KO mice (*p* < 0.05) (Fig. 6C & D). It is worth noting that although *Znt*8KO mice had reduced plasma insulin levels in both fasting condition and during OGTT, we did not detect any changes in peripheral insulin sensitivity in *Znt8*KO mice in both dietary groups compared to the WT controls (Fig. 6E & F).

### Weight gains and adiposity of a double knockout for *Znt8* and *Sst* mice

**Fig. 7:**
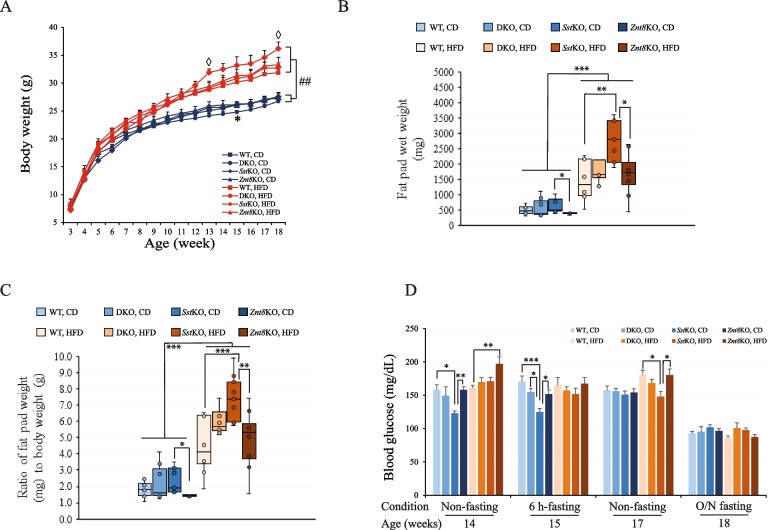
Body weight, fat pad weight, and blood glucose levels in male WT, DKO (double knockout for *Znt8*KO and *Sst*KO), *Znt8*KO and *Sst*KO mice. **A **Growth curve. The CD-fed DKO mice had significantly lower weight than the WT control at 15 weeks of age (*, *p* < 0.05). All four genotypic mice gained significant weight after 15 weeks on HFD compared to the respective CD group (##, *p* < 0.01). In addition, among the HFD challenged groups, the *Sst*KO mice were significantly heavier than WT at 12 and 18 weeks of age (◊, *p* < 0.05). **B **Fat pad wet weight (mg). The HFD-fed mice accumulated significantly more fat than the CD-fed counterparts in all genotypic groups (**, *p* < 0.01; ***, *p* < 0.001). In the CD-fed groups, *Znt8*KO mice had the least amount of fat than the other three genotypic mice (*, *p* < 0.05). **C** The ratio of fat pad weight to body weight. The HFD-fed mice had increased ratios of fat pad weight to body weight compared to the CD-fed counterparts in all genotypic groups (**, *p* < 0.01; ***, *p* < 0.001). In the CD-fed groups, *Znt8*KO mice had the lowest fat to weight ratio than the other three genotypic mice (*, *p* < 0.05). **D** Fasting and non-fasting blood glucose levels over the dietary challenge period. *, *p* < 0.05; **, *p* < 0.01; ***, *p* < 0.001. All data are presented as mean ± S.E., *n* = 7–10/group. Body weight, fat pad weight, and blood glucose data were analyzed by a one-way or two-way ANOVA test to compare the means of multiple groups when appropriate followed by a post-hoc test (Tukey’s test). CD, chow diet; HFD, high-fat diet

Considering the detection of colocalization of ZnT8 with Sst in the gastrointestinal D cells, we posited that a compensatory mechanism involving Sst might be critical for responsiveness to *Znt8* deficiency in the normalization of glucose metabolism. Thus, to investigate the interaction of ZnT8 and Sst in the control of glucose metabolism, we generated a double knockout mouse line for *Znt8* and *Sst* (DKO). We performed metabolic phenotyping related to insulin levels, glucose handling, and obesity in male DKO mice along with single knockout of *Znt8* or *Sst* mice and the WT control. We conducted a 15-week feeding study as depicted in Fig. 5A. Regardless of genotypes, all mice fed HFD gained significantly more weight than their counterparts maintained on CD (*p* < 0.01). The difference became evident starting at 8 weeks of age, which corresponded to 5 weeks on HFD (*p* < 0.01). Notably, after 11 weeks of HFD feeding, *Sst*KO mice started to gain significantly more weight than DKO, *Znt8*KO, or WT mice (*p* < 0.05) (Fig. 7A).

Increased visceral adipose depot is a central feature of metabolic syndromes that relates to systemic inflammation in humans [42]. In mice with diet-induced obesity, the visceral fat primarily consists of epididymal and retroperitoneal adipose tissues, which can be assessed by the total weight of the visceral fat and the ratio of fat weight to body weight at necropsy. As shown in Fig. 7B, HFD feeding significantly increased the wet weight of visceral fat depots in all genotypic mice (*p* < 0.001). Among the four genotypes in the HFD groups, *Sst*KO mice accumulated the most visceral fat compared to the other three genotypes (36.7% more than DKO, *p* > 0.05; 160% more than *Znt8*KO, *p* < 0.05; and 86.5% more than WT, *p* < 0.01). It is worth noting that the CD-fed *Znt8*KO mice also presented a significantly reduced visceral fat mass compared to *Sst*KO mice in the same dietary group (*p* < 0.05) (Fig. 7B). Similar results were obtained from the assessment of the ratio of visceral fat weight to body weight (Fig. 7C). In the HFD-fed groups, *Sst*KO mice were the most obese compared to the *Znt8*KO and WT mice (*p* < 0.01 compared to *Znt8*KO; *p* < 0.001 compared to WT). Again, *Znt8*KO mice were leaner than *Sst*KO mice in the CD group (Fig. 7B). Taken together, these findings suggest that *Sst* knockout alone greatly increased body weight and adiposity in mice challenged with HFD.

### Fasting and non-fasting blood glucose levels in DKO mice

To investigate the effect of DKO on glucose metabolism during a 15-week HFD challenge, we monitored blood glucose levels under fasting or non-fasting condition and compared them to the controls, including WT, *Znt8*KO, and *Sst*KO mice (Fig. 7D). Non-fasting blood glucose concentrations were assessed at 14 and 17 weeks of age (11 and 14 weeks after introduction of HFD, respectively). Fasting glucose levels were measured at 15 (6-h fasting) and 18 weeks old (overnight fasting). As shown in Fig. 7D, under the CD feeding condition, *Sst*KO mice had significantly lower non-fasting and 6-h fasting blood glucose levels than the other three genotypic mice (*p* < 0.05) at 14 and 15 weeks old, respectively. However, these differences were not detectable when mice were at 17–18 weeks age. When mice were challenged with HFD, only *Znt8*KO mice displayed a significant difference in non-fasting glucose levels compared to WT at 14 weeks of age (*p* < 0.01). Again, this difference was not detected when mice reached 17 weeks of age (Fig. 7D). Remarkably, at 17 weeks of age, *Sst*KO mice had significantly lower non-fasting blood glucose level than WT and *Znt8*KO mice (*p* < 0.05). Taken together, these results suggest that *Znt8*KO mice may be more while *Sst*KO may be less susceptible to the diet-induced abnormality in non-fasting glucose levels during HFD challenge among the four genotypic groups. In contrast, DKO mice had a minimal impact on blood glucose levels in both CD and HFD feeding conditions when compared to WT.

### Impaired peripheral insulin sensitivity in DKO mice after high-fat diet challenge

**Fig. 8:**
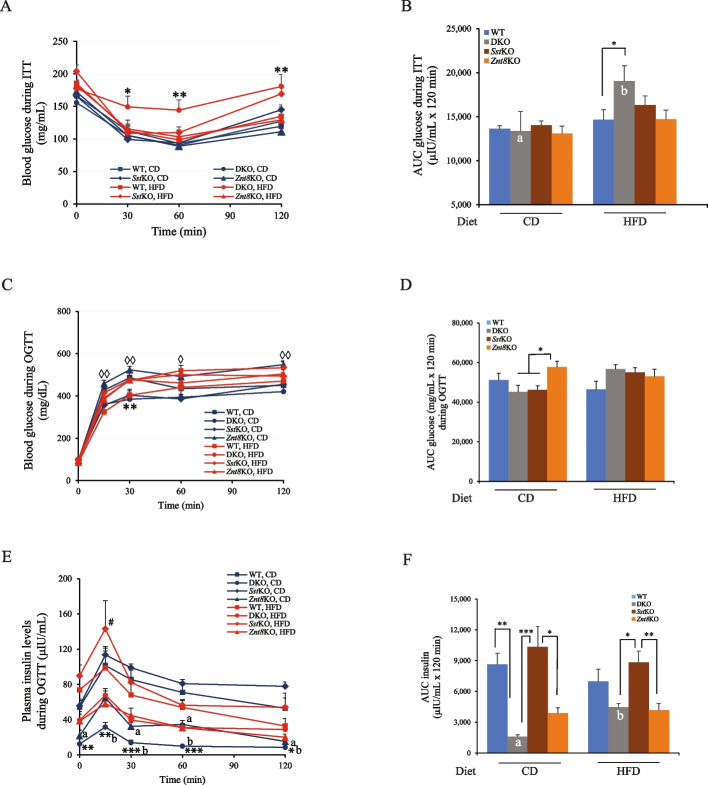
ITT and OGTT in male DKO, *Znt8*KO, *Sst*KO, and WT mice. **A **Blood glucose levels during ITT. A significantly increased blood glucose levels were observed in the HFD-fed DKO mice at 30, 60 and 120 min post the insulin stimulation compared to the WT and *Znt8*KO mice in the same dietary group (*, *p* < 0.05). **B **AUC of blood glucose levels during ITT. The * denotes the significant difference between DKO and WT mice in the HFD groups as indicated (*, *p* < 0.05). Different letters (a and b) indicate statistical significance between the two dietary groups with the DKO genotype (*p* < 0.05). **C **Blood glucose levels during OGTT. DKO mice fed CD had a significantly decreased blood glucose level at 30 min (**, *p* < 0.01) post the glucose stimulation compared to the WT control. The postprandial glucose levels at 15, 30, 60, and 120 min were all significantly lower in the CD-fed DKO mice than the CD-fed *Znt8*KO mice (◊, *p* < 0.05; ◊◊, *p* < 0.01). **D **AUC of blood glucose levels during OGTT. A significantly decreased AUC was observed in the CD-fed DKO and *Sst*KO mice compared to *Znt8*KO mice in the same dietary group (*, *p* < 0.05). **E **Plasma insulin levels during OGTT. The * denotes the CD-fed DKO mice had significantly lower plasma insulin levels at the baseline and 15, 30, 60, and 120 min after glucose challenge than the WT control or *Sst*KO (*, *p* < 0.05; **, *p* < 0.01; ***, *p* < 0.001). The letter a implies the significant difference between the *Znt8*KO and WT or *Sst*KO mice in the CD group (*p* < 0.05). The letter b shows the significant difference between DKO and *Znt8*KO mice in the CD groups (*p* < 0.01). The # denotes the significant difference between DKO and *Sst*KO mice in the HFD groups (*p* < 0.01). **F **AUC of plasma insulin levels during OGTT. The * denotes the significant difference between genotypic groups as indicated (*, *p* < 0.05; **, *p* < 0.01). Different letters (a and b) indicate statistical significance between the two dietary groups with the DKO genotype (*p* < 0.05). All data are presented as mean ± S.E., *n* = 8–10/group. Blood glucose or plasma insulin data was analyzed by a one-way or two-way ANOVA test to compare the means of multiple groups when appropriate followed by a post-hoc test (Tukey’s test). CD, chow diet; HFD, high-fat diet

To determine the effect of HFD feeding on insulin sensitivity in the peripheral tissues of DKO mice, we conducted insulin tolerance test (ITT) at 16 weeks of age. As shown in Fig. 8A & B, no significant changes in peripheral insulin sensitivity were detected in all genotypic mice fed CD. However, the HFD-fed DKO mice had severe impairment in glucose disposal compared to WT at 30, 60, and 120 min, to *Sst*KO at 30 and 60 min, and to *Znt*8KO at 30, 60, and 120 min after an intraperitoneal insulin injection (Fig. 8A). The AUC of blood glucose level over the 120 min post-insulin injection period in the HFD-fed DKO mice was increased by ~ 30% compared to WT and *Znt8*KO mice (*p* < 0.05) (Fig. 8B). However, no significant diet-induced insulin resistance was observed in *Sst*KO and *Znt8*KO mice when compared to WT (*p* > 0.05).

### Impaired oral glucose tolerance in the CD-fed DKO mice

Next, we examined the response to an oral glucose administration after 15 weeks on either CD or HFD (18 weeks old). As shown in Fig. 8C, we detected a clear peak of a glucose surge at 30-min time point after the oral glucose load in the CD-fed WT and *Znt8*KO mice, whereas this glucose surge was blunted in the CD-fed DKO and *Sst*KO mice (*p* < 0.01). The AUC of blood glucose level over the 120 min post-glucose period in the CD-fed DKO and *Sst*KO mice was ~ 22% lower than the CD-fed *Znt8*KO mice (*p* < 0.01) (Fig. 8D). However, there were no significant differences in the AUC of glucose levels between WT, and *Znt8*KO within the CD groups. Nevertheless, no significant increase in the AUC of glucose levels was noted in the HFD-fed DKO mice compared to other three genotypes in the same dietary group (Fig. 8D). Together, these results suggest that a postprandial glucose surge in the blood requires Sst secretion.

### Impaired insulin secretion in DKO mice

To further investigate the impact of DKO on insulin secretion, we quantitated blood insulin levels during OGTT. As shown in Fig. 8E, under the CD-feeding condition, DKO mice had the lowest overnight fasting insulin levels (µIU/mL) among the four genotypic mice (DKO, 12.6 ± 0.5, *n* = 6; WT, 54.1 ± 9.2, *n* = 6; *Sst*KO, 55.7 ± 7.5, *n* = 8; *Znt8*KO, 21.9 ± 6.2, *n* = 6). Glucose-stimulated insulin secretion at 30, 60, and 120 min was also significantly impaired in the CD-fed DKO during OGTT compared to WT, *Sst*KO, or *Znt8*KO (*p* < 0.01). The AUC of insulin levels during the 2-h course of OGTT were significantly reduced in the CD-fed DKO mice compared to both WT and *Sst*KO mice in the same dietary group (*p* < 0.01 and *p* < 0.001, respectively) (Fig. 8F). The AUC of insulin levels also decreased during the 2-h course of OGTT in the CD-fed *Znt8*KO mice compared to the CD-fed *Sst*KO mice (*p* < 0.05), despite being in a smaller degree than that of DKO mice (Fig. 8F). Similar results of glucose-stimulated insulin secretion and the AUC of plasma insulin concentrations during OGTT were obtained from the four genotypic mice in the HFD groups. Additionally, the HFD-fed DKO mice had an increased insulin secretion at all time points during OGTT compared to the CD-fed DKO (*p* < 0.01) (Fig. 8F). However, no significant difference in glucose-induced insulin secretion was observed between *Sst*KO and WT mice under either dietary condition. Taken together, *Znt*8KO negatively affects glucose-induced insulin secretion regardless of diets and DKO enhances this negative effect in the mice fed CD.

**Fig. 9:**
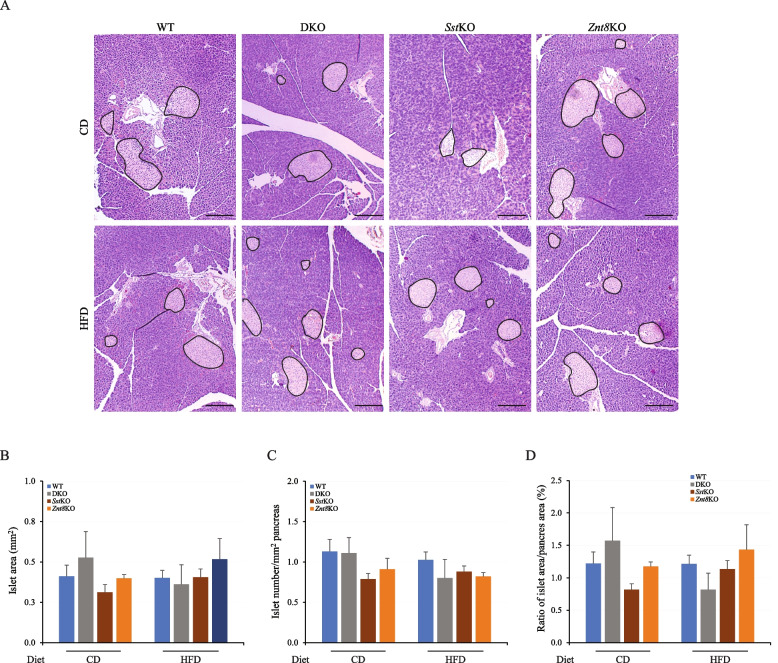
Histological staining of the pancreatic tissue, area of islets (mm^2^), numbers of islets per mm^2^ of the pancreas, and the ratio of the area of islets to the area of the pancreas in male mice fed either CD or HFD diet. Mice were euthanized at 18 weeks of age after 15 weeks on the indicated diet. **A **H&E staining of the pancreas tissues from WT, DKO, *Sst*KO, and *Znt*8KO mice. **B **Islet area (mm^2^). **C **Islet number/pancreas area (mm^2^). **D **Ratio of islet area (mm^2^)/pancreas area (mm^2^). Scale bar is 200 µm. The islets in the pancreas were circled in black. CD, chow diet; HFD, high-fat diet; WT, wild-type; DKO, double knockout for *Sst* and *Znt8*; *Sst*KO, *Sst* knockout; *Znt8*KO, *Znt8* knockout. All data are presented as mean ± S.E., *n* = 3–11/group. CD, chow diet; HFD, high-fat diet

A pronounced reduction in glucose-stimulated insulin secretion in the CD-fed DKO mice prompted us to examine islet areas, numbers, and the ratio of islet area to the pancreas area in DKO mice, comparing them to the other three genotypic mice. As shown in Fig. 9A, no significant difference in islet sizes, islet area to the pancreas area or morphology of the islets was noted between DKO and WT, *Sst*KO, or *Znt8*KO mice in either dietary group. These findings suggest that the pronounced impairment of insulin secretion found in the DKO mice was not associated with structural alterations in the pancreatic islet.

### Effect of DKO on plasma metabolic-related hormone levels

**Fig. 10:**
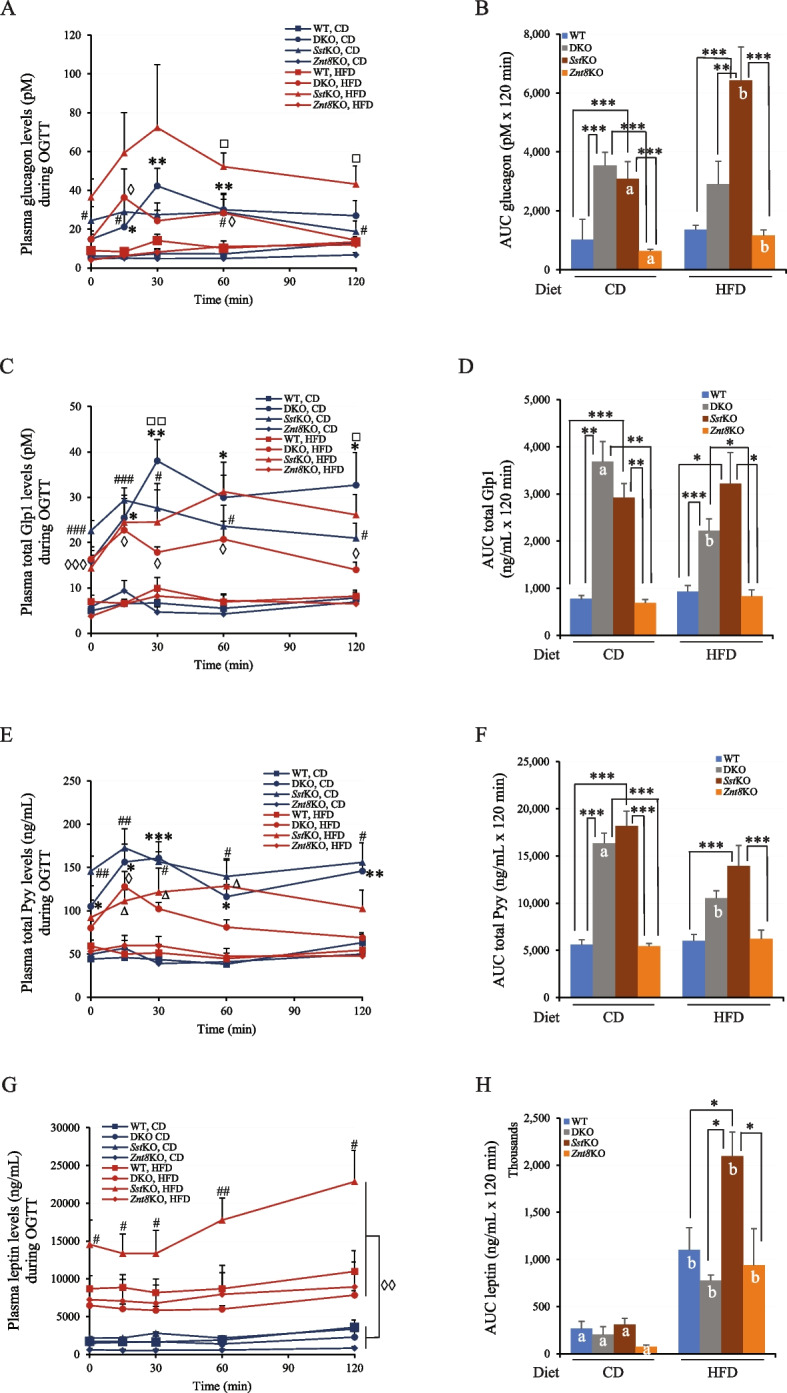
Plasma glucagon, Glp1, Pyy, and leptin levels during OGTT in male mice fed either the chow diet or high-fat diet. **A **Glucagon levels. The * denotes the significant difference between DKO and WT or *Znt*8KO in the CD group (*, *p* < 0.05; **, *p* < 0.01). The # implies the significant difference between the *Sst*KO and WT or *Znt8*KO in the CD groups (*p* < 0.05). The ◊ denotes the significant difference between DKO and WT or *Znt8*KO in the HFD dietary group (*p* < 0.05). The □ shows the significant difference of *Sst*KO between the CD and HDF groups (*p* < 0.05). **B **AUC of plasma glucagon levels. **C **Glp1 levels. The * denotes the significant difference between DKO and WT or *Znt*8KO in the CD group (**, *p* < 0.01). The # implies the significant difference between the CD-fed *Sst*KO and the WT or *Znt8*KO in the same dietary group (#, *p* < 0.05; ###, *p* < 0.001). The ◊ denotes the significant difference between DKO and WT, *Sst*KO, or *Znt8*KO in the HFD dietary group (◊, *p* < 0.05: ◊◊◊, *p* < 0.001). The □ shows the significant difference of DKO between the CD and HDF groups (□, *p* < 0.05; □□, *p* < 0.01). **D **AUC of plasma Glp. **E **Pyy levels. The * denotes the significant difference between DKO and WT or *Znt*8KO in the CD group (*, *p* < 0.05; **, *p* < 0.01). The # implies the significant difference between the CD-fed *Sst*KO and the WT or *Znt8*KO in the same dietary group ((#, *p* < 0.05; ##, *p* < 0.01). The ◊ denotes the significant difference between DKO and WT, *Sst*KO, or *Znt8*KO in the HFD dietary group (◊, *p* < 0.05). The ∆ implies the significant difference between the HFD-fed *Sst*KO and the WT or *Znt8*KO mice in the same dietary group (*p* < 0.05). **F **AUC of plasma Pyy. **G **Leptin levels. The # implies the significant difference between the HFD-fed *Sst*KO and the WT, DKO, or *Znt8*KO in the same dietary group (#, *p* < 0.05; ##, *p* < 0.01). The leptin levels were significantly increased at all examined timepoints during OGTT in all genotypic groups after 15-weeks of HFD challenge compared to the respective levels in the CD groups (◊◊, *p* < 0.01). **H **AUC of plasma leptin. Panels B, D, F, and H, the * denotes the significant difference between groups as indicated (*, *p* < 0.05; **, *p* < 0.01; ***, *p* < 0.001). The groups with different letters indicate the significant difference between the two diets within the same genotype. All data are presented as mean ± S.E., *n* = 6–8/group. Data was analyzed by a one-way or two-way ANOVA test to compare the means of multiple groups when appropriate followed by a post-hoc test (Tukey’s test). CD, chow diet; HFD, high-fat diet

Given the potent inhibitory action of Sst on the endocrine system and the presence of ZnT8 in other endocrine cells other than β-cells, such as the D cells of the gastrointestinal tract (Figs. 1 & 2) and the α-cell of the pancreas [8], we examined the changes of other metabolic-related hormone levels in the circulation during OGTT, including glucagon (produced by the α-cell in the islet), Glp1 (produced by the L-cell in the intestine), leptin (Lep, produced by the adipocyte), and Pyy (produced by the L-cell in the ileum and colon). Glucagon raises glucose concentrations during fasting, its secretion suppressed by the elevated glucose level in the islet. Glucagon secretion is also negatively regulated by Sst. As expected, both CD-fed DKO and *Sst*KO mice displayed significantly elevated glucagon levels during OGTT compared to WT and *Znt8*KO mice fed the same diet (*p* < 0.05), suggesting a failure in glucose-induced suppression of glucagon secretion in DKO and *Sst*KO mice (Fig. 10A). Furthermore, when *Sst*KO mice were subjected to HFD, they presented additional impairment of glucagon suppression ((*p* < 0.05). The AUC of glucagon levels during OGTT was two-fold higher in the HFD-fed *Sst*KO mice than the CD-fed *Sst*KO mice (*p* < 0.05); while DKO and WT mice remained comparable AUC of glucagon levels between the two diets (Fig. 10B), suggesting that an additional knockout of *Znt8* on the *Sst*KO genetic background would alleviate the negative impact of *Sst*KO on the glucose-induced glucagon suppression. It is worth noting that the HFD feeding also increased glucagon secretion in *Znt8*KO mice compared to the same genotypic mice fed CD (*p* < 0.05) (Fig. 10B).

Glp1, known to promote insulin secretion in a glucose-dependent manner [43], was examined during OGTT in the study mice. As shown in Fig. 10C, like the secretion pattern of glucagon during OGTT, both DKO and *Sst*KO mice exhibited significantly higher plasma Glp1 levels than the WT and *Znt8*KO mice across all dietary conditions and at all examined time points during OGTT (*p* < 0.05). The AUC of plasma Glp1 levels during OGTT was 4.0-fold and 3.0-fold higher in DKO and *Sst*KO mice, respectively, than WT or *Znt8*KO in the CD group. (Fig. 10D). This pattern was persistent even when these mice were fed HFD, indicating a robust stimulation of Glp by glucose regardless of diet. Moreover, HFD challenge reduced Glp1 secretion by 40% in DKO mice during OGTT compared to DKO mice in the CD group (Fig. 10D). Nevertheless, HFD challenge seemed to have a minimal effect on Glp1 secretion during OGTT in WT, *Sst*KO, and *Znt8*KO mice compared to the same genotypic mice in the CD groups.

Secretion of Pyy is stimulated by food intake, inducing satiety [44]. Thus, we determined the circulating Pyy levels during OGTT in the study mice. The results showed that, in the CD-fed groups, both DKO and *Sst*KO mice displayed significantly higher Pyy levels than the WT and *Znt8*KO mice at all timepoints examined during OGTT (*p* < 0.01). This was also reflected in the AUC of plasma Pyy concentrations (Fig. 10F). We noted that the high-fat diet challenge seemed to have little to no effect on the Pyy levels in WT and *Znt8*KO mice (Fig. 10 E & F). We also noted that the HFD challenge did not further increase plasma Pyy levels in *Sst*KO mice during OGTT (Fig. 10 E & F). Whereas we observed that Sst knockout-induced elevation of Pyy levels was partially reversed in DKO mice evident by a 36% decrease of the AUC of plasma Pyy levels during OGTT (*p* < 0.05) (Fig. 10F).

Circulating leptin levels are positively correlated with the amount of body fat and reflect the amount of energy stored in fat and the acute changes in food intake [45, 46]. We then examined leptin levels during OGTT in the experimental mice. In the CD groups, no difference of plasma leptin levels during OGTT was found between DKO and WT mice (*p* > 0.05) (Fig. 10G and H). The HFD challenge greatly increased plasma leptin levels in all genotypic mice (*p* < 0.01). While the heightened leptin levels after HFD challenge were comparable among WT, DKO, and *Znt8*KO mice, the *Sst*KO mice had approximately twofold higher plasma leptin concentration by comparison (Fig. 10G & H). Taken together, the plasma leptin concentration was remarkably increased in the HFD-fed *Sst*KO mice before and after the glucose load. However, the presence of *Znt8*KO on the *Sst*KO genetic background ameliorated the increase in plasma leptin levels caused by the *Sst* knockout alone.

## Discussion

ZnT8 is a key player in both glucose [18] and lipid metabolism [47], with associations to risk of T2D in humans [9, 13]. Somatostatin plays a negative regulatory role in many metabolic-related hormones, including insulin and glucagon in the pancreas and GIP and GLP1 in the gastrointestinal tract. Hence, both ZnT8 and somatostatin are directly or indirectly involved in glucose and insulin metabolism. However, the phenotypic effects of *Znt8* and *Sst* interactions in regards of regulation of glucose homeostasis and its impact on the risk of chronic diseases, such as obesity and T2D, remain unknown. In the current study, we demonstrated that male *Znt8*KO mice fed CD had a similar growth rate with WT, consistent with previous findings [24, 47]. To our surprise, we noted that a 15-week HFD challenge did not induce extra weight gain in male *Znt8*KO mice compared to WT, contradictory with the observation made by Nicolson et. al. in the Toronto colony of *Znt8*KO [20]. In addition, we detected an elevated non-fasting glucose level in the HFD-fed *Znt*8KO mice at 14 but not 17 weeks old, indicating HFD induced a transient abnormality in glucose metabolism in the early stage of dietary challenge in *Znt8*KO mice, a compensatory mechanism being able to normalize glucose metabolism. Likewise, a transiently elevated fasting glucose levels was detected in male Toronto *Znt8*KO mice at 6 weeks of age, which normalized at 12 weeks old [20]. We also noticed that *Znt*8KO mice had decreased insulin secretion at both basal and glucose-induced conditions, regardless of diets. This is slightly different from the male Toronto *Znt8*KO mice in which basal insulin release was elevated accompanied by reduced glucose-induced insulin secretion [20]. Nevertheless, insulin sensitivity in the peripheral tissues of mice was not impacted by the *Znt8* knockout in this study as well as in others [19, 20, 47]. It is known that the phenotypic expressivity of *Znt*8KO in the regulation of glucose metabolism is dependent on the genetic background of mice. *Znt8*KO mice used in this study were on the genetic background of C57BL/6NJ while the Toronto colony of *Znt8*KO mice were on a mix of the genetic backgrounds of SV129 and C57BL/6J (three backcross of SV129 to C57BL/6J) [20]. Therefore, it is not surprising to see phenotypic variabilities among *Znt8*KO mice generated from different strains of mice [19, 20, 47].

Given that ZnT8 and Sst were co-expressed in the D cell of the mouse stomach and small intestine, the rat pancreatic D cells (Suppl. Figure 1) [48], and the increasing numbers of atypical dense cores in the secretory granules of the mouse *Znt8*^*−/−*^ D cells, we hypothesized that Sst might play an important role in the normalization of glucose metabolism in dietary challenged *Znt8*KO mice. This is by balancing glucagon, Glp1, and Pyy releases as Glp1 and Pyy are vital hormones in maintaining euglycemia by regulating insulin secretion, inhibiting glucagon secretion, reducing appetite, increasing satiety, and delaying gastric emptying [49, 50]. Furthermore, Sst regulates these hormones through its paracrine (SS-14) or endocrine (SS-28) action to inhibit their secretion [51–53]. In this study, we showed that male *Sst*KO mice were susceptible to diet-induced obesity with increased visceral fat accumulation, consistent with the findings reported by Luque et al. [54]. We noted that the increase in weight or fat accumulation in *Sst*KO mice was ameliorated by DKO. On the contrary, DKO further impaired glucose-induced insulin secretion when mice were maintained on the chow diet without apparent effect on glucose levels compared to the *Znt8*KO mice. Reduced insulin sensitivity was also obvious in the HFD-fed DKO mice compared to the other controls (WT, *Sst*KO, and *Znt8*KO). These findings highlight the intricate relationship between ZnT8 and Sst in regulating insulin secretion. Altogether, our findings suggest that normalization of glucose metabolism in *Znt8*KO mice requires Sst as well as an intact condition in insulin sensitivity.

SST is initially produced as a preprosomatostatin protein with a signal peptide at the N-terminal end (1–24 amino acids), which is cleaved in the ER forming a prosomatostatin peptide. The SS-14 and SS-28 peptides are then produced by proteolytic cleavage of the prosomatostatin by two prohormone convertases, PC1 (generating SS-28) and PC2 (generating SS-14) during maturation and processing through the Golgi apparatus to the secretory granules. PC1 and PC2 are also involved in processing proinsulin and proglucagon in the islets of the pancreas [55]. Both PC1 and PC2 are Ca^++^-dependent enzymes, their activity stimulated by a lower pH around 5.5–6.0 [56]. Studies from insulin-secreting granules of β-cells suggest that zinc transport via ZnT8 to granules was coupled to alkalinization of vesicles through the Zn^++^/H^+^ coupled anti-transporting mechanism [57]. It can be speculated that, in the Sst-expressing D cells, a reduction in ZnT8 activity caused by either ZnT8 allelic deficiency or by *Znt8*-null mutations would decrease pH in granules due to reduced Zn^++^/H^+^- coupled antiport activities. This subsequently stimulates the activities of PC1 and PC2 resulting in an increased prosomatostatin cleavage. As the result, over-produced active forms of Sst would cause self-aggregation forming atypical dense particles instead of forming a rather homogenous matrix found in the secretory granules of the WT D cells (Fig. 4) [58]. It is plausible to assume that the increased density in the dense cores of the secretory granules of the *Znt8*KO D cell was the result of an enhanced production of Sst in response to the absence of ZnT8.

Predictably, the glucose-induced glucagon suppression was impaired in the HFD-fed *Sst*KO mice (Fig. 10) as Sst secreted from neighboring D cells is required for the glucose-induced inhibition of glucagon release from α-cells [38, 50]. Solomou et. al. reported that ZnT8 is required for hypoglycemia induced glucagon secretion from α-cells in the islets without significantly affecting glucose tolerance in the α-cell-specific *Znt8* KO mice [8]. Our results support this notion. It is well known that glucagon secretion is elevated in T2D leading to the release of stored glucose from the liver to the circulation that exacerbates the disease condition. Results from this study indicate that the excessive release of glucagon induced by *Sst*KO and HFD challenge was significantly inhibited by the absence of ZnT8 expression, suggesting that reduced ZnT8 expression is favorable for controlling glucose levels by inhibiting excessive glucagon release. This observation is consistent with the findings in humans that rare null mutations (ZNT8 allelic deficiency) are protective for T2D development [13, 17].

In addition, both DKO and *Sst*KO mice presented significantly higher levels of fasting or fed (during OGTT) Glp1 and Pyy than *Znt8*KO or WT mice regardless of diets, consistent with the inhibitory function of Sst on the secretion of these hormones. Given that ZnT8 is not co-expressed with Glp1 in the gastrointestinal tract (Fig. 2), it was surprising to observe that the HFD-fed DKO mice also had significantly reduced Glp1 and Pyy releases during OGTT compared to *Sst*KO mice. ZnT8 has not been reported to play a role in regulating Glp1 or Pyy secretion in humans or mice. More research is needed to determine the functional link between ZnT8 and Glp1 or Pyy in control of glucose metabolism.

Leptin, a hormone secreted from adipocytes [59], is crucial for regulating long-term energy balance to maintain energy reserves through storage of triglycerides in adipocytes [60]. In this study, we showed that the HFD-challenged *Sst*KO mice were extremely obese (Fig. 7B and C) and had markedly increased fasting and fed (during OGTT) blood leptin levels compared to the other three genotypic mice. This was likely caused by the increased amount of adipose tissue in *Sst*KO mice [60]. Interestingly, double knockouts of *Znt8* and *Sst* led to alleviation of escalated leptin levels found in *Sst*KO mice after HFD challenge. This remarkable change in leptin level was accompanied by a significantly decreased body fat accumulation in DKO mice fed HFD. A plausible explanation of this phenotype is that ZnT8 is involved in fat storage in adipose tissue either directly or indirectly. This assumption is supported by the association of the human ZNT8 protein with postprandial blood triglyceride clearance after a high-fat mixed macronutrient challenge in men and obese individuals [61]. However, the functional interaction between ZnT8 and triglyceride storage or leptin secretion in adipose tissue with the regulation of energy balance has not been previously reported. Further investigations are needed to address ZnT8 activity in triglyceride metabolism and to adopt ZnT8 as a useful gene target in studying precision nutrition or precision medicine for obesity and T2D prevention or treatments.

## Conclusion

In the current study, we demonstrated that ZnT8 is co-expressed with Sst in a subpopulation of endocrine D cells of the gastrointestinal tract and the absence of ZnT8 expression resulted in an increased density of dense cores in the secretory granules of the D cell. We showed that ZnT8 is required for excessive secretion of glucagon, Glp1, Pyy, and leptin during high-fat diet challenge in *Sst*KO mice. We also showed that Sst is needed for glucose-stimulated insulin secretion during OGTT. Finally, we demonstrated that ZnT8 may have a role in regulating fat mass and leptin secretion. These findings shed light on the multifaceted nature of ZnT8 and Sst interactions. This study opens a new avenue for understanding the roles of ZnT8 and Sst in controlling glucose metabolism, paving the way for potential therapeutic interventions type 2 diabetes.

## Supplementary Information

Supplementary Material 1.

## Data Availability

No datasets were generated or analysed during the current study.
